# The role of trust, expectation, and deception when buying organic animal products

**DOI:** 10.1093/af/vfac080

**Published:** 2023-02-23

**Authors:** Sarah Kühl, Elisa Bayer, Maureen Schulze

**Affiliations:** Department of Agricultural Economics and Rural Development, University of Göttingen, Göttingen, Germany; Department of Agricultural Economics and Rural Development, University of Göttingen, Göttingen, Germany; Department of Management, Society and Communication, Copenhagen Business School, Frederiksberg, Denmark

**Keywords:** animal welfare, consumer behavior, consumer deception, organic, trust

**Implications**

- Consumer trust is highly important for the success of organically certified animal products.
- Becoming aware of the gap between individual expectations and the reality on farms can cause distrust and a feeling of deception among consumers.
- The feeling of deception does not automatically reduce consumers’ intention to buy organic products—one reason is that organic products are still perceived as the best option for animal welfare.
- As the expectation–reality gap is widening, the organic sector should use the time available to adapt handling conditions in organic farming to align with consumer expectations.

## Introduction

**Figure 1.**
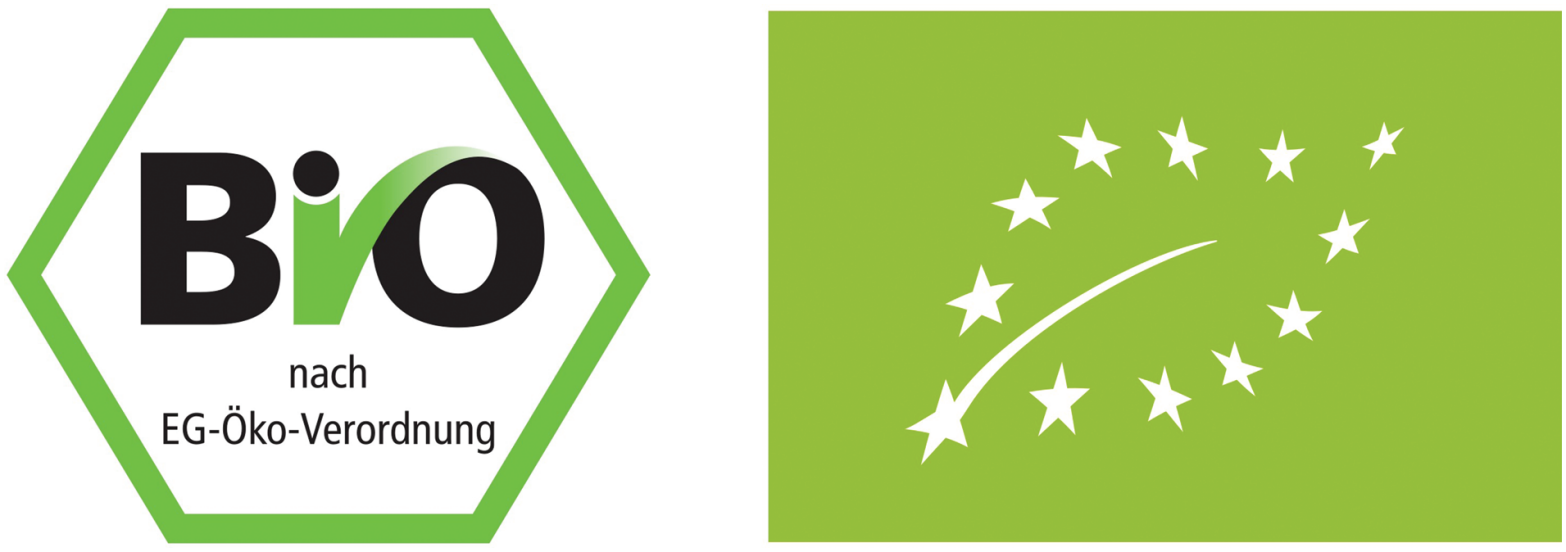
German (left) and European (right) organic label; source: https://www.oekolandbau.de/bio-siegel/.

Over the last years, consumer interest in how livestock is raised and handled and thus how animal-based products such as milk, meat, and eggs are produced, has increased in the Western world (Alonso et al., 2020). As a result, several market-driven initiatives tackling consumer demand for products produced with higher animal welfare standards were introduced (Esbjerg et al., 2022). Nowadays, consumers are confronted with a variety of front-of-package food labels such as the “Beter Leven” label in the Netherlands or the “Initiative Tierwohl” in Germany. The most well-known and widespread label associated with animal welfare from a consumer’s perspective is the organic label (Schleenbecker and Hamm, 2013; Meyer-Höfer et al., 2015; Figure 1): more than 95% of German consumers are familiar with the German organic label (Zühlsdorf et al., 2016) and the more animal-friendly husbandry systems are the most important buying motive for organic meat consumers (Zander and Hamm, 2009).

Labels, such as the organic label, are needed to help consumers make purchasing decisions according to their preferences. Without these labels, consumers would not be able to identify how animal-based products were produced as the keeping of animals is a so-called “credence attribute” that cannot be verified on the product itself (Darby and Karni, 1973). Consumers neither have the possibility nor the expertise to control the promised product characteristics, such as keeping and feeding of the animals. Therefore, trust in the label and certification is crucial when buying organic products (Nuttavuthisit and Thøgersen, 2017). Previous research has shown that higher levels of trust are associated with higher confidence that the products meet the promised standards (Wu et al., 2021). In contrast, distrust lowers the expectations and thus makes it less likely that consumers buy a labeled product (Nuttavuthisit and Thøgersen, 2017). To guarantee that products adhere to the promised standards, and thus increase consumer trust, credible and understandable third-party certification schemes are often used to monitor the production process (Alonso et al., 2020).

**Figure 2.**
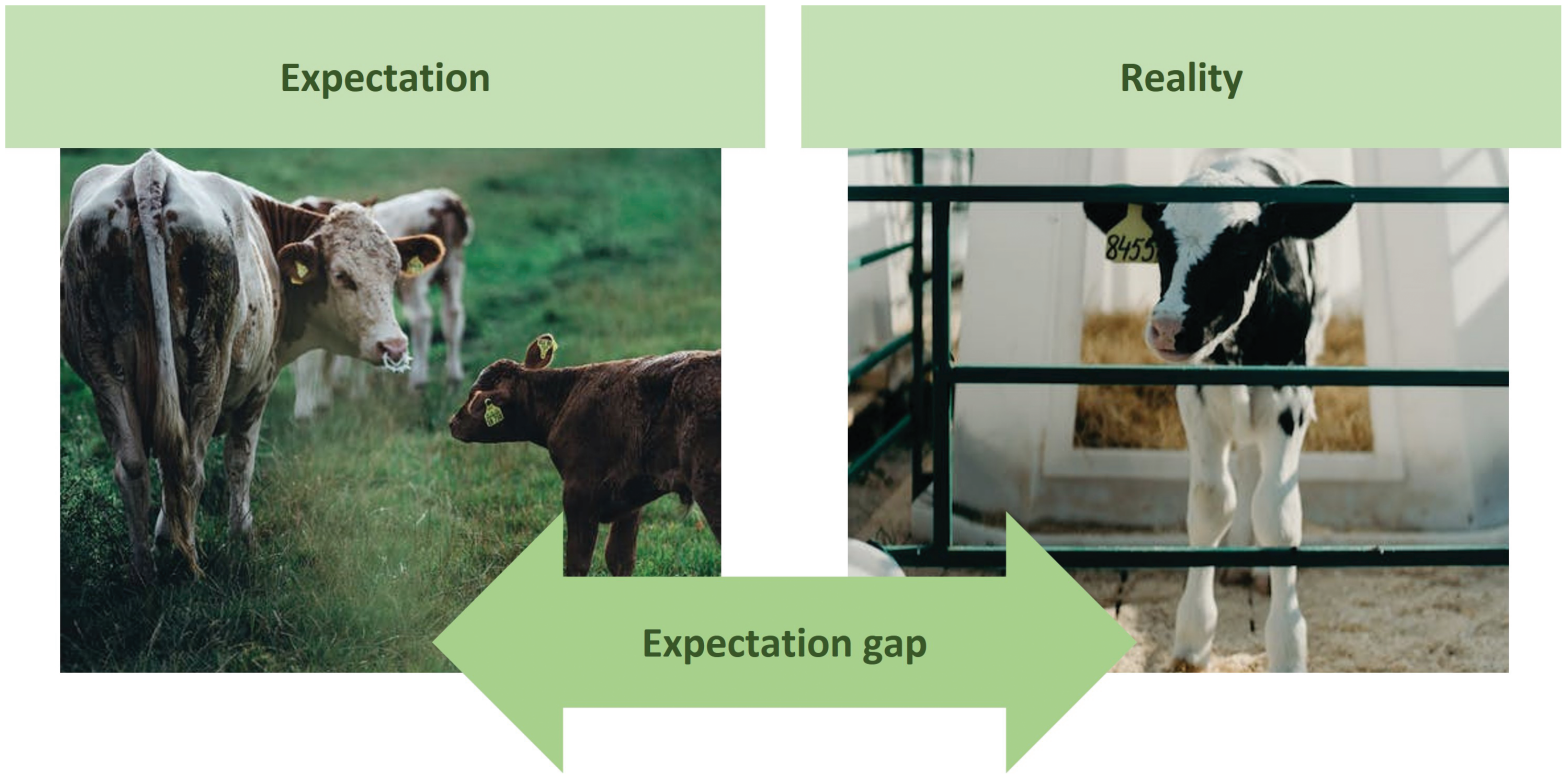
Expectation gap on the example of calf rearing; own illustration.

Such labels serve to inform consumers about the standards behind them in a simplified way as consumer knowledge about livestock farming is limited (Di Pasquale et al., 2014). The low level of knowledge along with positive perceptions of organic can lead consumers to have false associations or overly high expectations of organic animal products and the respective husbandry systems (Hermansen, 2003). Von Meyer-Höfer et al. (2015) conclude in their study that, from an overarching perspective, there is not a large gap between consumers’ expectations of organic products and the actual reality. However, the authors further point out that less is known regarding consumers’ expectations about specific animal welfare issues and a possible gap between expectation and reality, which could potentially lead to consumer deception. The practice of early cow-calf separation is a suitable example to investigate whether consumer expectations of organic farming exceed reality on farms, whether this evokes a feeling of deception, and how the feeling of deception influences consumer behavior. So far, early cow-calf separation is common in conventional but organic dairy farming (Kälber and Barth, 2014). However, most citizens are not aware of this procedure and prefer that calves are raised by their mothers (Hötzel et al., 2017). The appearing gap between consumer expectation and reality (expectation gap, Figure 2) could, if revealed, have the potential to reduce trust in the organic label.

Thus, in the following, next to findings from existing studies to emphasize this topic’s relevance, new consumer study results will be presented to investigate how consumers cope with disenchanted perceptions of organic products and how this affects their future buying behavior.

## The Expectation Gap and its Consequences

As consumers are not able and do not have the expertise to prove whether the organic label fulfills the promised certification criteria, for example, higher animal welfare standards (=“credence attribute” as mentioned above), they have to trust the information provided on the product. Tonkin et al. (2015) summarize the relevance of trust for food labels as follows: “Trust is needed where there is no assurance, trust is the bridging of uncertainty and lack of knowledge; trust is vulnerability.” Existing studies reveal that trust is one of the most important influencing factors for consumers’ buying intention and willingness to pay higher prices for organic products (Canova et al., 2020). Nocella et al. (2010) conclude that trust in stakeholders’ compliance with certification standards for animal welfare products is essential for consumers to decide to buy these products. A loss of trust, meanwhile, leads to a lower willingness to buy (Canova et al., 2020). Thus, trust in those products is highly important for the market success of organic labels.

Additionally, trust in credence attributes is strongly linked to expectations. Nocella et al. (2010) more precisely argue that trust “is based on the expectation that the trustee (e.g., the producer or retailer) will perform a particular action important to the trustor (the consumer), irrespective of the ability of the latter to monitor or control such an activity”. These findings illustrate that trust is very vulnerable to false expectations. If the expectations consumers have of a particular label exceed the reality, or consumers become aware that a label does not meet their expectations, they may feel disappointed or even deceived. Additionally, literature in the field of psychology confirms that unmet expectations lead to a loss of trust (Möllering, 2008).

At the same time, consumers’ expectations toward animal welfare standards of the organic label are based on their (often low) knowledge and the information given on the product. Thus, without further information, there is some space for interpretations and expectations that exceed the real organic criteria. Additionally, consumers search for and concentrate on positive aspects to justify their buying decisions and to gain a positive feeling. This effect is particularly apparent in the case of higher-priced products (Gelderman et al., 2018), such as organic products. Organic meat is on average two to three times more expensive than conventional meat in Germany (Schaack et al., 2018), and the price is mentioned as the main barrier for buying organic meat (Padel and Foster, 2005). Thus, organic consumers focus on the benefits (e.g., increased animal welfare or health value) of organic production to justify their purchases of those more expensive products (Padel and Foster, 2005). Furthermore, it is known that the organic label often acts as a heuristic cue to a general superiority of organic products. This phenomenon is called “halo-effect” and can lead to the fact that consumers portray the organic sector as more attractive than it actually is and suppress thoughts about aspects that, in their eyes, do not fit the organic sector (von Meyer-Höfer et al., 2015). Therewith, the already mentioned gap between expectations toward the organic label and the real criteria might increase.

To bring expectations closer to reality, information can be helpful. According to the information-deficit-model, filling consumers’ knowledge gap has an influence on subsequent behavior. However, inconsistent scientific results exist on how additional information on livestock farming influence consumers’ meat consumption behavior: While some information may increase acceptance of certain production processes, in other cases, it may have the opposite effect and consumers become more critical with more information (Kwasny et al., 2022). Ruhnke et al. (2010) add that expectation gaps are not only based on missing information but also appear due to unfulfilled but reasonable expectations. Nevertheless, it is known that transparency plays a key role when it comes to trust while marketing animal welfare-certified products (Nocella et al., 2010). Not least because consumers themselves have little knowledge e.g., of how farm animals are actually kept (Di Pasquale et al., 2014). Cornish et al. (2020) showed that providing additional information about the benefits of a label significantly increased the intention to buy animal welfare products.

However, when it comes to buying decisions, it must further be distinguished between cognitive and affective behavior. While cognitive decisions are based on rational considerations, information, and knowledge (e.g., comparing price and performance), affective decisions are more emotional (e.g., feeling good about the purchase; Trandafilovic et al., 2013). It is known that the decision to buy organic products can be both cognitive and affective (Padel and Foster, 2005). In addition, this distinction can also be made between cognitive and affective trust. While some consumers base their trust on knowledge and careful consideration, others focus more on trust and feelings (Lahno, 2013). For people who favor cognitive trust, information and knowledge are more important and have a greater impact on their buying decision than for people who base their buying decision more on emotional trust (Lahno, 2013). Further, there is habitual trust which is based on routine and habit, meaning consumers trust a product or label because they have had repeated satisfying experiences and little disappointment with the product or label (Delhom, 2013).

Thus, it becomes evident that expectations are important to generate and maintain trust and to avoid consumers being disappointed or even feeling deceived when marketing credence-based products such as organic products. Especially, as disappointed expectations might lead to a reduced willingness to buy and pay for higher-priced animal welfare products (Thorsøe, 2015).

In the following, we will discuss the current literature on trust in organic products and the impact of disappointed expectations. However, as scientific findings on this topic are rare, we add some findings from our own consumer study of 574 German meat consumers that was conducted in 2022. This study aimed to investigate consumers’ reactions and the feeling of deception when confronted with a possible reality-expectation gap. As an example of a situation in which consumers might have expectations and preferences that deviate from reality, the practice of early cow-calf separation in organic farming was chosen as example. Most consumers expect that organic animals are kept under more animal-friendly conditions (Schleenbecker and Hamm, 2013), and the separation of cow and calf directly after birth is considered less animal friendly from a consumers’ perspective (Busch et al., 2017). Thus, the early cow-calf separation could make most consumers feel deceived when they are confronted with the fact that a practice that is known to be rejected by a large share of people is also allowed in organic dairy production, and thus it might not meet their expectations (“Expectation gap”, Figure 1; Thorsøe, 2015; Kühl et al., 2017).

**Figure 3.**
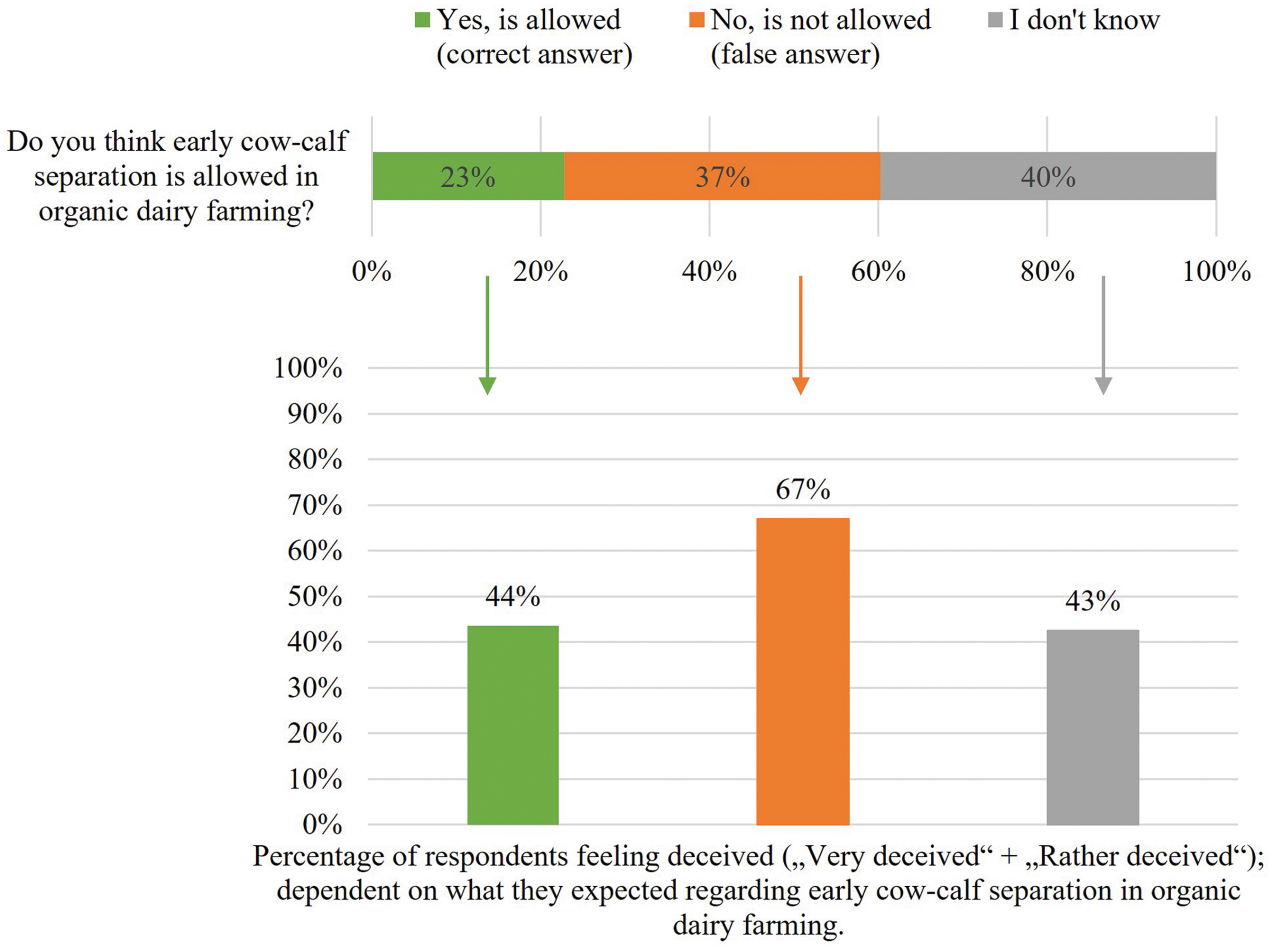
Respondents’ expectations toward the question of whether the early cow-calf separation is also allowed in organic dairy farming and the percentage of respondents who feel deceived after being informed that it is allowed in organic dairy farming dependent on what they expected; question for the second figure: “Do you feel deceived by the regulation that early cow-calf separation is also allowed in organic milk production*?*”, shown are Top boxes (“Very deceived” + “Rather deceived”, on a 5-point Likert-scale); *n* = 574; own figure.

In our study, participants were informed that in dairy farming, calves are usually separated from their mothers immediately after birth and were asked if they believed that this is also allowed in organic dairy farming. The results show that a large percentage of respondents (40%) were unsure whether the early separation of calves and cows is also allowed in organic dairy farming. This underlines existing research showing the low knowledge of consumers when it comes to animal husbandry (Di Pasquale et al., 2014). However, 37% expected that the early separation is not allowed in organic dairy farming, and significantly fewer correctly assumed that it is allowed (23%; Figure 3, upper part).

Afterward, all respondents were asked whether they felt deceived by the fact that the early separation of calves and cows is also allowed in organic dairy farming. Overall, the majority of respondents stated that they felt deceived (52%), 34% felt at least partly deceived, and only 14% did not feel deceived (not shown in figures). It is noticeable that people who had indicated in the previous question that they assumed that the early separation of cow and calf is not allowed in organic farming were more likely to feel deceived when they were informed about it: 67% of these respondents felt deceived, while only 44% of those who expected correctly that the practice is also common in organic farming and 43% of those who indicated that they did not know felt deceived (Figure 3, lower part). It is quite interesting that those, who stated that they do not know whether the early separation of calves and cows is also allowed in organic dairy also tend to feel less deceived, similar to those who correctly expected this procedure to be allowed. This underlines the significant role of expectations when it comes to the feeling of deception and, therewith, to trust: with correct or no expectations the risk of feeling deceived is decreased (Nocella et al., 2010).

**Figure 4.**
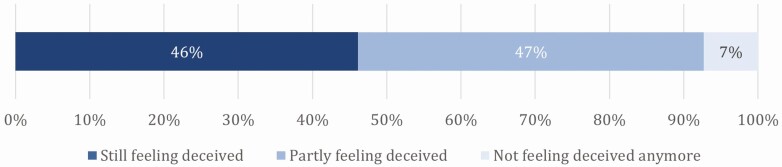
Respondents’ feeling of deception after being informed that the information about the regulation of early cow-calf separation is available in the internet; question: “Information about early cow-calf separation in organic animal husbandry can be found transparently on the internet. Do you still feel deceived?”, *n* = 493; own figure.

In a next step, we informed those consumers who stated to feel deceived (*n* = 493) that the information about this regulation is available on the internet and asked them whether they still felt deceived. The aim of this question was to find out whether consumers’ sense of deception can be reduced once they know that the information is publicly available and thus that there is not an actively or intentionally induced deception. The results show that only 7% changed their opinion and stated to (rather) not feel deceived anymore when becoming aware of the fact that this information is transparent and available online. For 46%, the awareness that the information is available transparently does not lead them to feel less deceived; for an equally large proportion, the feeling of having been deceived decreased at least somewhat (Figure 4).

The results shown in Figures 3 and 4 confirm existing studies that false expectations or perceptions lead to a higher sense of deception when confronted with them (e.g., von Meyer-Höfer et al., 2015; Kühl et al., 2017). This seems obvious, since one’s own expectations are not met, and a feeling of disappointment is in most cases produced by unmet expectations (Held and Germelmann, 2019). It is interesting to note, however, that even people who correctly suspected that the early separation of calves and their mothers is permitted in organic animal husbandry still felt deceived to a large extent. Even the clarification that the information about the actual practices is available on the internet is not enough to significantly reduce this sense of having been deceived. This underlines the finding of Ruhnke et al. (2010) that expectation gaps are not necessarily due to a lack of information but also to expectations that are reasonable from the consumer’s point of view.

**Figure 5.**
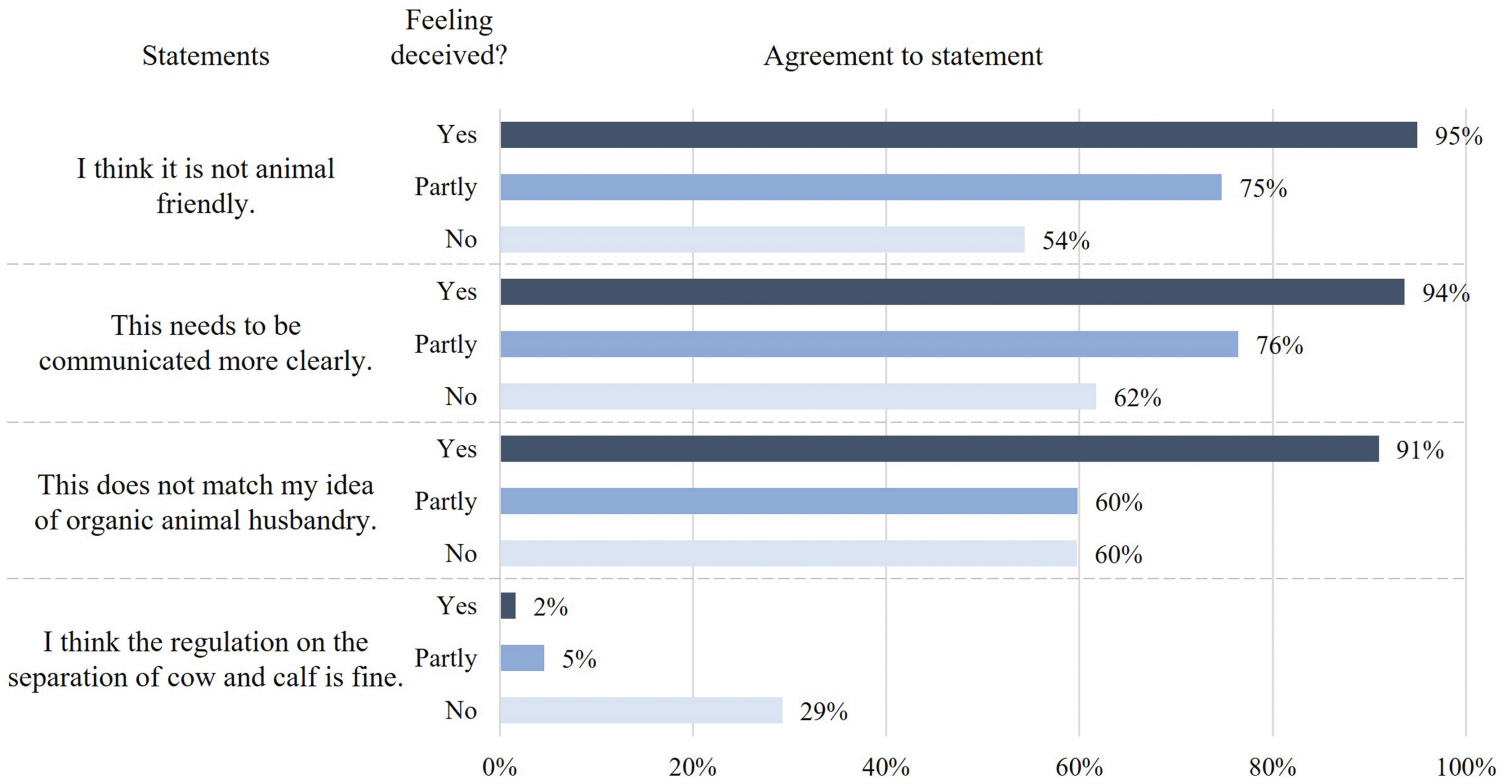
Respondents’ agreement to the four shown statements depending on the extent to which they felt deceived; shown are Top boxes (“Totally agree” + “Agree”, on a 5-point Likert-scale); *n* = 574; own figure.

The results in Figure 5 provide insights into some underlying reasons for consumers’ feeling of deception. Consumers who felt deceived agreed more than the other groups that the procedure is not animal friendly (95%), needs to be communicated more clearly (94%), and does not correspond to their idea of organic animal farming (91%). Thus, the reasons seem to be twofold: On the one hand, consumers expectation toward organic animal husbandry does not fit with the early cow-calf separation, on the other hand, the active information of consumers about this fact is criticized. The latter issue in combination with the result that the enlightenment about the fact that information on this procedure can be found on the internet underlines that this type of information is not in line with consumers’ expectations toward transparent information. Existing research confirms that consumers prefer on-package information (Alonso et al., 2020). However, the first aspect of unfulfilled expectations again emphasizes the statement by Ruhnke et al. (2010) that expectation gaps can also be rooted in expectations that consumers have about particular products. The reasons behind such expectations can be manifold. In this case, it seems to be a strong rejection of the early separation of cow and calf (Busch et al., 2017; Hötzel et al., 2017). Participants who did not feel deceived showed a clearly higher acceptance of the practice to separate calves and cows directly after birth (29%). However, the results that mainly those who feel deceived agree more with the statements confirm the relationship between expectations and the feeling of deception.

Therefore, we can summarize that consumers’ feelings of deception might be associated, at least among others, with the following factors: whether they consider the practice to be (not) animal friendly, whether they expect it to be commonly done in organic dairy farming, and how they assess the respective information to be (actively) communicated. The mere existence of information on the internet does not seem sufficient to make consumers feel less deceived. However, Busch et al. (2017) and Hötzel et al. (2017) found that even information about the reasons why calves are separated from their mothers at an early stage does not increase the acceptance of this practice. This shows that such strong attitudinal patterns cannot be overcome easily. Thus, from the results shown so far and from the existing literature, we can conclude that there will be practices that, even with an explanation, communication, and transparency, will be rejected by the majority of consumers and may lead to a feeling of disappointment when associated with the organic industry, which in turn may affect the trust in the organic sector(Thorsøe, 2015).

Therewith consumers’ trust in organic products can be threatened by the feeling of disappointment, which appears when consumers are confronted with the reality that may not meet their expectations (Tonkin et al., 2015). Further also the current regulations of the organic industry themselves can already cause disappointment and thus are a trust issue in the long run. This may be the reason why even those people who correctly expected early cow-calf separation to be allowed in organic livestock farming were disappointed when it was confirmed.

**Figure 6.**
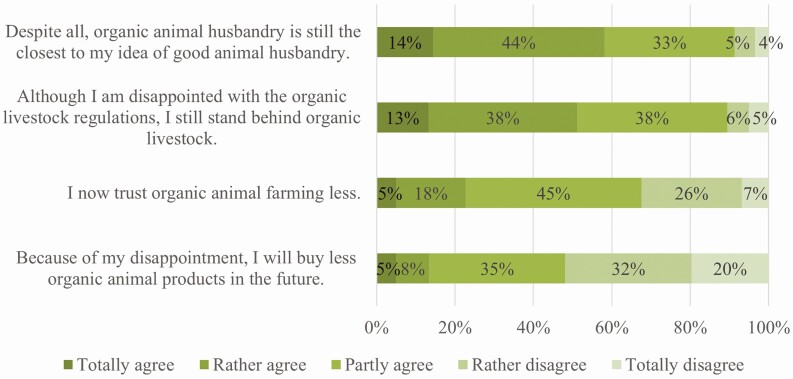
Respondents’ agreement to the shown statements; only respondents who stated that they felt deceived by the regulation that early cow-calf separation is also allowed in organic dairy farming were asked to rate their agreement; *n* = 493; own figure.

The question of whether consumers’ trust will also be permanently damaged and how it might affect the willingness to buy organic products is still unanswered. Therefore, all respondents who stated that they felt deceived were asked whether they trusted organic livestock farming less due to their disappointment, whether they would buy fewer organic products in the future, or whether organic was still the best choice for them. Figure 6 shows that the majority of respondents agreed that for them organic animal farming is still the best option (58%) and, although they might be disappointed that calves are separated from their mothers directly after birth in the organic sector, they would still support it (51%). However, at least 23% agreed that the awareness of this procedure lowered their trust in organic animal farming, and 13% stated that they would buy fewer organic products. We further checked if these attitudes differed between consumers who buy organic meat more often and those who felt more deceived, but there were no significant differences. Our results cannot confirm that those individuals who consume organic products more often are more likely to feel deceived or that such a feeling leads to a greater loss of trust or has a higher impact on the likelihood of purchasing organic products in the future. However, our results suggest that this is mainly due to the fact that organic is currently (still) perceived as the best alternative. This highlights the risk to the organic sector if there are other labels or certifications that precisely target and advertise certain consumer concerns, as is already being done with products from farms where mother-bonded calf rearing is practiced.

## Conclusion

Over the years, organic farming has become a well-known and highly trusted certification system that stands for a high level of animal welfare. The positive image seems to compensate for recognizing that individual expectations exceed the reality on organic farms. Nevertheless, it would be wrong to assume that no action from the organic sector is required to reduce the gap between consumer expectations and reality. As the existence of information on the internet, and therewith the possibility of consumers becoming aware of real practices, does not reduce the majority’s feeling of having been deceived, only providing more information about organic farming practices would not necessarily reduce the degree to which consumers might feel deceived when getting aware that not accepted practices are also common in the organic sector. More, so far, it is unknown to what extent the positive image of the organic label can compensate for negative information. Additionally, research analyzing consumers’ expectations and assessments of specific practices related to animal welfare is rare. The organic sector should use the available time to get more insights into consumers’ expectations and demands toward organic animal husbandry and adapt handling practices that are in line with consumer expectations. However, there are as yet no findings on how the confrontation with negative information affects trust in the long term. In general, studies show a clear correlation between disappointed expectations and reduced trust. The fact that this is less the case with the present results on trust in organic animal products, when confronted with negative information, might be due to the fact that there are hardly any alternatives to organic products so far and organic is seen by many as a premium standard and heuristic cue for an overall better product (“halo-effect”). However, if products with even higher standards are introduced in the near future, the market pressure will be increased.

## About the Authors

**Dr. Sarah Kühl** studied business administration at Leuphana University of Lüneburg (Germany) and then worked in sales. This was followed by a master’s degree in equine science and a doctorate on the topic of “Differentiation strategies based on agricultural production processes in the food market” at the Chair of Marketing for Food and Agricultural Products, University of Göttingen (Germany). Following the PhD, Sarah Kühl took up a position as a research assistant at the Chair of Livestock Sciences at the Free University of Bolzano (Italy) and conducted, i.a., economic analyses of livestock farms. After working as a strategic consultant, she returned to the Chair of Marketing for Food at the University of Göttingen as a postdoc and is working on consumer behavior in organic and animal welfare products as well as local and digital marketing of food.

**Elisa Bayer** is a PhD student at the Chair of Marketing for Food and Agricultural Products at the University of Göttingen (Germany). She studied “organic agriculture” (bachelor and master level) at the University of Kassel/Witzenhausen (Germany). Her main focus was on a sustainable agriculture, animal welfare, and consumer behavior. After her master’s, she joined the doctoral program at the University of Göttingen and is currently working on a project which investigates consumers’ perceptions and expectations of organic animal husbandry and which aims to increase social acceptance of these more animal-friendly husbandry systems. Her research interests are in animal-friendly husbandry systems and consumer behavior.

**Maureen Schulze** is a postdoctoral researcher at the Department of Management, Society and Communication at Copenhagen Business School (Denmark). She studied agricultural economics (B.Sc. and M.Sc.) in Kiel (Germany) and got her PhD degree from the University of Göttingen (Germany). During her doctoral studies, she investigated consumer demands and food retailers’ marketing strategies to foster a more sustainable livestock sector. Her current research focuses on the green transition of the food and agricultural system, particularly on interventions for consumers’ preferences and attitudes and involved stakeholders to help build a sustainable food system.
